# Supplementary material for: Combinatorial Effect of Cold Atmosphere Plasma (CAP) and the Anticancer Drug Cisplatin on Oral Squamous Cell Cancer Therapy
Source: Int J Mol Sci. 2020 Oct 15;21(20):7646. doi: 10.3390/ijms21207646 (PMC7588898; doi:10.3390/ijms21207646)
Supplement: Supplementary file 1 [file ijms-21-07646-s001.pdf]

Supplementary Materials

Methods

Live and death cell staining of HGF-1 and SCC-15 cells

1×10^5 cells in 12 wells were treated with cisplatin and/or CAP as same as described in MTT assay. Briefly, cells were treated with cisplatin for 6h followed by CAP treatment. Then, cells were washed with PBS twice and then incubated for 15 min with 1 mL of Live/Dead cell staining solution (1 µl live dye (1mM) and 1 µl dead dye solution (1mg/mL propidium iodide) (PI) in staining buffer 1ml) at 37 °C. Following this, cells were observed with fluorescence microscope under dark condition (Eclipse Ni-U, Nikon, Tokyo, Japan).

Results

As shown in Figure s1, HGF-1 cells showed higher density of live cells as a green dye compared to SCC-15 cells while SCC-15 cells showed higher density of dead cells as a red dye. These results support the results of Figure 4.

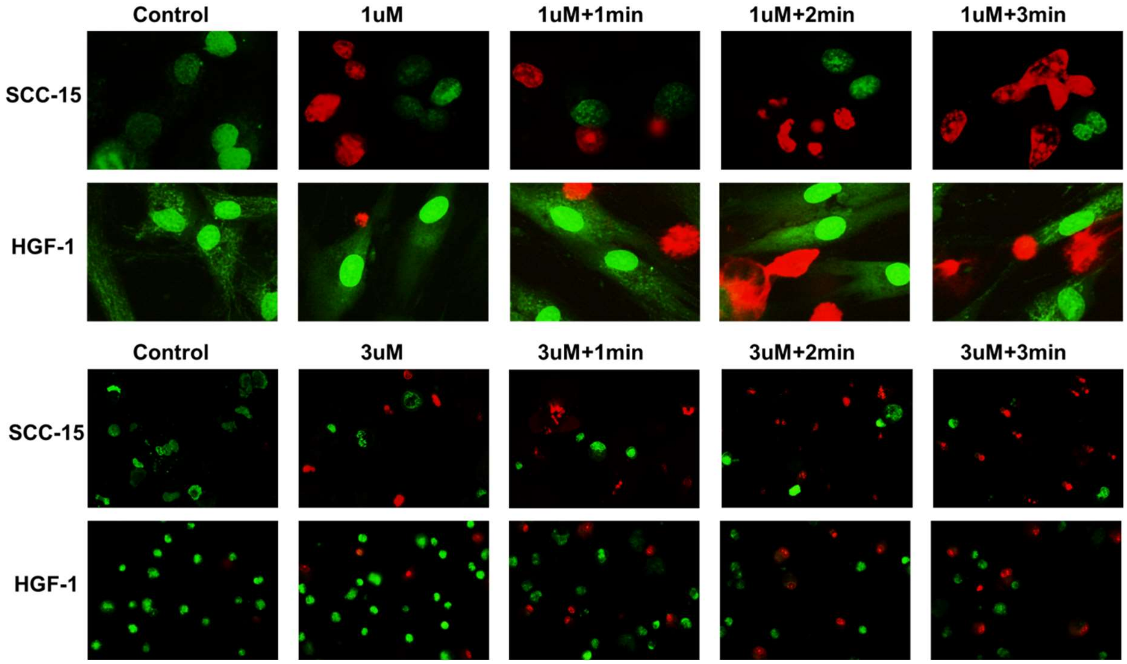

Figure S1. Live and dead cell staining of cells. Cisplatin and CAP were treated to SCC-15 cells and HGF-1 cells as described in Figure 4. Cells treated with cisplatin/CAP were stained with Live/Dead cell staining solution. Green and red color represents live and dead cells, respectively.
